# Centring human health in the global plastics treaty: a call to action

**DOI:** 10.1136/bmjgh-2022-011040

**Published:** 2022-11-14

**Authors:** Megan Deeney, Joe Yates, Rosemary Green, Suneetha Kadiyala

**Affiliations:** Department of Population Health, London School of Hygiene and Tropical Medicine Faculty of Epidemiology and Population Health, London, UK

**Keywords:** Environmental health, Public Health

**Summary box**

- Negotiations for a global treaty to end plastic pollution present a distinct window of opportunity for the global health research and practice community to shape lasting policy that delivers for people and planet.
- Evidence exists for diverse human health impacts throughout the plastic life cycle, but quantifying these effects is challenging, making health, and health equity concerns vulnerable to exclusion from political debate and to false claims from vested interests.
- Global health researchers and practitioners should be prepared, not only to supply evidence of the health risks of plastics and waste reduction strategies, but also to adjudicate claims of plastic health benefits made throughout negotiations.
- The global health community must collaborate to generate and synthesise evidence on complex pathways between plastics, waste reduction strategies and health, ensuring effective communication of knowledge through an overarching, inclusive, council for health that addresses the full life cycle of plastics.

Plastics are not only implicit in a planetary pollution crisis, they are damaging ecosystems and responsible for the burning of ever-increasing amounts of fossil fuels, driving biodiversity loss and climate change in ways that are less discussed, but equally alarming.^1^ Linked to these forces is a growing recognition of both direct and indirect human health impacts throughout the plastic life cycle, stimulating recent calls for greater engagement from the public health community.^2^ The question is: are we building evidence on health effects fast enough? Negotiations for a global treaty to end plastic pollution aim to establish a legally binding international agreement by 2024.^3^ This is a narrow but distinct window of opportunity for the diverse global health research and practice community to converge and collaborate, engaging with a true planetary health emergency that demands a united, global health response.

Awareness of possible human health impacts of pollution is not new, but for plastics, official recognition still often amounts to little more than a nod. Health hazards of ocean pollution were already alluded to in the 1972 Stockholm Declaration, the first set of principles for global collaboration on environmental issues.^4^ Exactly 50 years on, the connection specifically between plastic pollution and human health is inconsistently recognised. The United Nations Environment Programme (UNEP) points to health effects in the resolution for a global plastics treaty and in a new evidence summary, 3 5 but more than half of published government statements supplied to inform the upcoming treaty negotiations make no reference to health at all (figure 1).^3 6^ In the same 50 years, the world has amassed around 7 billion metric tonnes of plastic waste, with up to 12 million tonnes pouring into the ocean each year.^7 8^ Though the treaty promises a monumental win for the environment, why has political discussion of the health impacts of plastics not advanced further?

**Figure 1:**
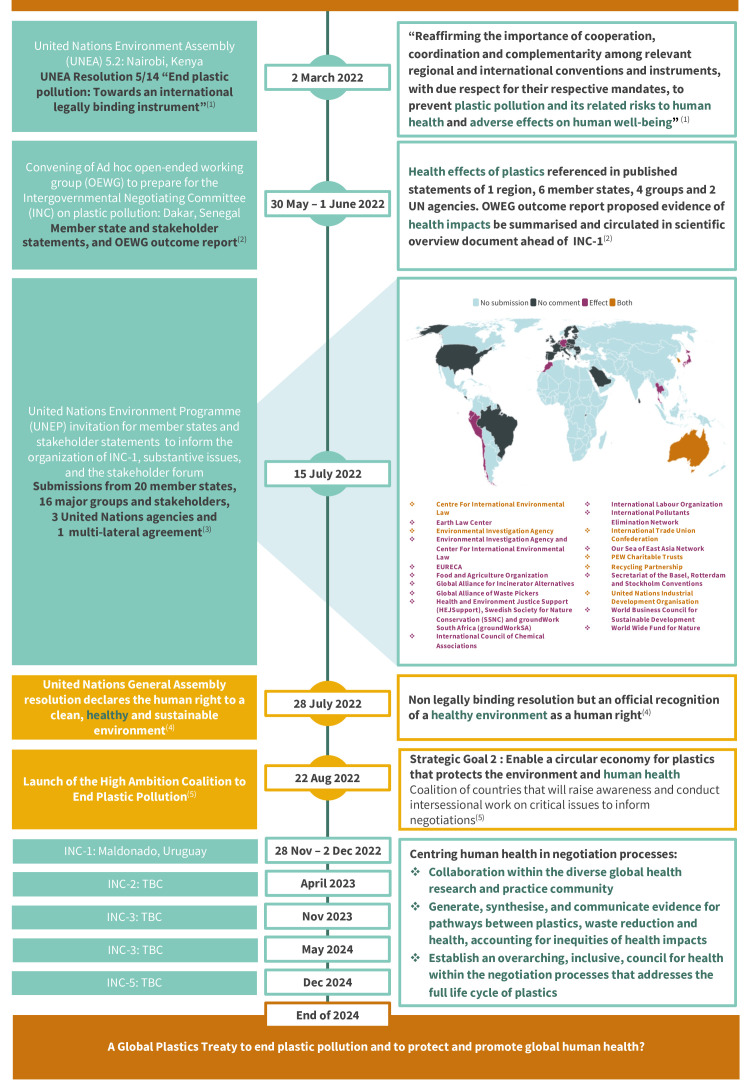
Timeline of the inclusion of global health considerations in key publications and milestones leading up to the start of negotiations for a global treaty to end plastic pollution. Notes: The figure includes references to and extracts from the following sources: (1) the United Nations Environment Assembly Resolution 5/14,^5^ (2,3) the United Nations Environment Programme (UNEP) webpages on the Ad hoc open-ended working group and the first meeting of the Intergovernmental Negotiation Committee,^3 6^ (4) the United Nations General Assembly Declaration on the human right to a clean, healthy and sustainable environment^12^ and (5) the High Ambition Coalition to End Plastic Pollution website.^13^ The geographical map visualises references to health as identified in country (map) and stakeholder (list) statements and references supplied to the UNEP in response to the invitation for comments ahead of the first meeting of the Intergovernmental Negotiating Committee.^3^ Light blue indicates no submission from a given country, dark grey indicates a submission but no mention of health, purple indicates the mention of health as an effect of plastics, and orange indicates inclusion of health as an effect of plastics and as a driver of plastic pollution, for example by specific mention of healthcare or personal hygiene related plastic products.

One reason may be the limited availability of scientific evidence that can be readily used to inform policy. Statements submitted to the UNEP by several non-governmental organisations summarise current evidence for aspects of human health effects including neurotoxicity, endocrine disruption, reproductive issues, respiratory problems, inflammation, increased cancer risk and damages to mental health as a result of pollutants released throughout the plastic life cycle (figure 2).^3 6^ However, quantification of these effects remains relatively scarce, particularly on the scale set by a global treaty, and generating this much-needed evidence is challenging.^9^ In research design terms, exposure to plastic covers an amorphous and evolving collection of different polymers, containing untold quantities of obscure chemicals. Controlling for a substance that is now ubiquitous in land, air and water is extremely difficult, as is accounting for combined and compounded pathways and measuring health effects that may be latent or even intergenerational. Though the profile of the treaty may encourage more studies, greater engagement and coordination within the health research community is needed,^2^ to streamline evidence generation with the urgency of effectively informing negotiations on health. In other words, we need to do in less than 2 years what we have not yet achieved in more than 50.

**Figure 2:**
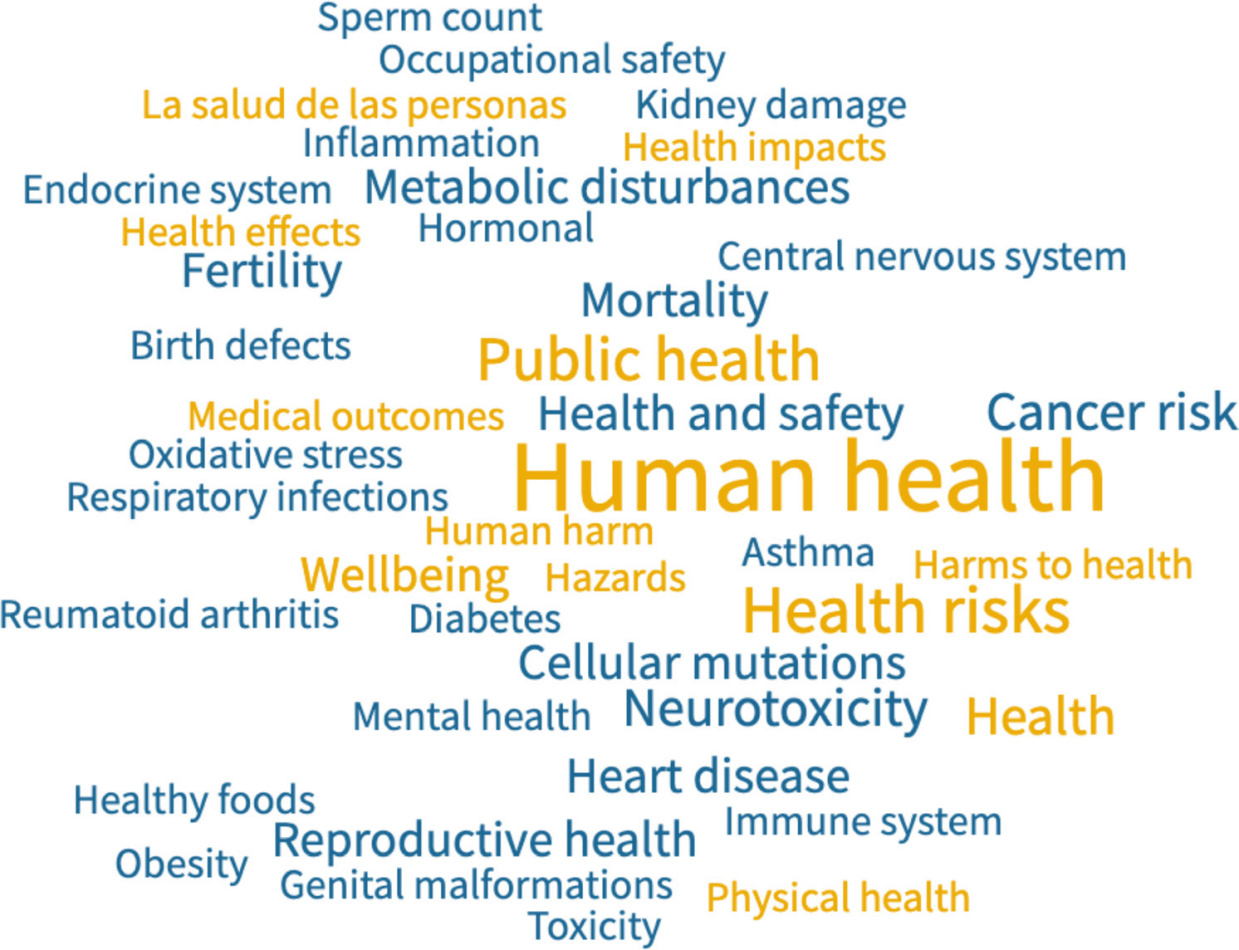
Word cloud of health-related terminology identified in country and stakeholder statements on the global plastics treaty, supplied to the United Nations Environment Programme (UNEP) ahead of the first meeting of the Intergovernmental Negotiating Committee.^3^ Notes: All identified health-related phrases have been included, the font size reflects relative frequency in the use of given phrases across statements. Yellow colour highlights general references to health, blue indicates specific health conditions and damages. 3

Where policy may be hindered by evidence and science may be hindered by methodological challenges, there are those that stand to benefit from uncertainties in health impacts. Fossil fuels and climate are often discussed separately to plastics and pollution, but these planetary issues, and the powerful industries driving them, are inextricably intertwined.^1^ Plastics remain almost exclusively derived from fossil fuels, and plastic production alone is set to generate 2.8 gigatons of greenhouse gas emissions per year by 2050.^1^ The oil and chemical industries behind plastic production share a history of obscuring knowledge of their detrimental impacts; where Exxon already knew about climate change in 1977, Monsanto was aware of adverse human health effects of Polychlorinated Biphenyls, used in the production of the plastic Polyvinyl Chloride, as early as 1930.^10 11^ Ahead of the plastics treaty negotiations, vested interests are barely concealed in certain statements that use health claims as a lever to maintain the status quo of plastic production. Saudia Arabia, Entidades Unidas Reafirmando la Economía Circular en Argentina (an industry-based group in Argentina) and the International Council of Chemical Associations with the World Plastics Council make unreferenced statements that plastics are important, even vital, to achieving the Sustainable Development Goals, that plastics protect human health, improve medical outcomes and access to healthy foods. These claims are paired with explicit statements that there should be no imposed bans, limits or controls on plastic production in the future global agreement.^3^ Plastics may well offer important benefits in some applications, but it is critical that we challenge reductive statements and legacy assumptions, and demand that any proposed benefits to society, should be demonstrated through evidence. To this end, global health researchers should be prepared, not only to supply evidence of risk but to adjudicate claims of benefits made throughout negotiations.

The human right to a healthy environment has now been officially recognised by the United Nations,^12^ and newly established groups such as the High Ambition Coalition to End Plastic Pollution are centring human health in their strategic goals^13^ (figure 1). However, in the development of the global treaty, human health arguments are being used in both in the prosecution and the defence of plastic, precipitating an even more urgent role for health researchers and professionals as we move into official negotiations. We need to generate, synthesise and communicate evidence on complex pathways between plastics, waste reduction strategies and health, accounting for the vast inequities in health impacts whereby the most vulnerable and least responsible for plastic waste and pollution, are likely to suffer the most.^14^ This is no small task and requires an overarching, inclusive, council for health that addresses the full life cycle of plastics, ensuring that this treaty will not shift the problem out of sight, to other sections of the life cycle, to other materials with unknown effects, to other countries or communities, or to other facets of health consequences. The global plastics treaty is a true test of the capacity and power of the global health research and practice community to reach across disciplines, collaborating and innovating evidence generation to ensure that the treaty protects both people and planet. So are we up to the challenge?

## Data Availability

All reviewed documents are available in full through the United Nations Environment Programme webpages, extracted information used in this article can be made available upon request to the corresponding author.
